# Health technology assessment of medical devices: current landscape, challenges, and a way forward

**DOI:** 10.1186/s12962-022-00389-6

**Published:** 2022-10-05

**Authors:** Jian Ming, Yunzhen He, Yi Yang, Min Hu, Xinran Zhao, Jun Liu, Yang Xie, Yan Wei, Yingyao Chen

**Affiliations:** 1 Real World Solutions, IQVIA, Shanghai, 200124 China; 2 National Health Commission Key Laboratory of Health Technology Assessment, School of Public Health, Fudan University, Shanghai, 200032 China

**Keywords:** Health technology assessment, Medical devices, Challenges, Real-world evidence, Review

## Abstract

**Background:**

Health Technology Assessment (HTA) has been widely recognized as informing healthcare decision-making, and interest in HTA of medical devices has been steadily increasing. How does the assessment of medical devices differ from that of drug therapies, and what innovations can be adopted to overcome the inherent challenges in medical device HTA?

**Method:**

HTA Accelerator Database was used to describe the landscape of HTA reports for medical devices from HTA bodies, and a literature search was conducted to understand the growth trend of relevant HTA publications in four case studies. Another literature review was conducted for a narrative synthesis of the characteristic differences and challenges of HTA in medical devices. We further conducted a focused Internet search of guidelines and a narrative review of methodologies specific to the HTA of medical devices.

**Main body:**

The evidence of HTA reports and journal publications on medical devices around the world has been growing. The challenges in assessing medical devices include scarcity of well-designed randomized controlled trials, inconsistent real-world evidence data sources and methods, device-user interaction, short product lifecycles, inexplicit target population, and a lack of direct medical outcomes. Practical solutions in terms of methodological advancement of HTA for medical devices were also discussed in some HTA guidelines and literature.

**Conclusion:**

To better conduct HTA on medical devices, we recommend considering multi-source evidence such as real-world evidence; standardizing HTA processes, methodologies, and criteria; and integrating HTA into decision-making.

**Supplementary Information:**

The online version contains supplementary material available at 10.1186/s12962-022-00389-6.

## Introduction

Health Technology Assessment (HTA) is a multidisciplinary process that uses a number of methods to determine the value of health technologies at different stages of their life cycle. HTA aims to provide evidence for health policy decision-making and for establishing an equitable, efficient, and high-quality health system [1]. Since its first application in the United States in the 1970s, HTA has developed rapidly and has been applied globally, becoming the basis for health decisions such as pricing and reimbursement in many different countries and regions. However, more of the existing HTA research concerns medicines rather than medical devices. Medical devices differ considerably from drug therapies in terms of their product lifecycle, regulatory environment, diversity, user–device interaction, and so on [2]. Even within medical devices, there are significant differences between therapeutic, instrumental, and diagnostic products. Moreover, various studies have investigated how these differences have posed great challenges to the HTA of medical devices and have thus called for applying a more innovative approach to medical devices compared to drugs. However, few studies have offered practical or actionable solutions. There is still a lack of consensus on the HTA of medical devices with regard to dimensions, process, criteria, and methods.

This study aimed to (1) describe the current landscape of HTA activities specific to medical devices; (2) analyze the characteristics of medical devices and the resulting challenges in the HTA of medical devices compared to pharmaceuticals; (3) perform a focused search of websites of official HTA agencies to identify international HTA guidelines specific to medical devices, intending to summarize implementable solutions to the HTA of medical devices. In addition, we supplemented the analysis of HTA guidelines with a narrative review of existing studies discussing the challenges of, and potential suggestions for, the HTA of medical devices.

## Method

To understand the landscape for HTA conducted on medical devices, we performed a retrospective analysis using IQVIA’s HTA Accelerator Database (www.iqvia.com/landing/hta-accelerator). It contains over 33,000 HTA publications that cover 100 HTA bodies in 40 countries. The primary data source came from the HTA submissions that could be tracked by local language. Market access experts from IQVIA were responsible for regularly tracking and translating all newly published HTA reports. The database captured over 250 available data elements such as the general information in the HTA report, including publication country, agency, publication date, disease area, product types, comparators, recommendations, etc. In this study, we focused only on HTA reports specific to medical devices in the HTA Accelerator Database by selecting the product type as “medical device.” We limited the assessment type of HTA submissions to health technology assessment or rapid review (including the assessment of safety, efficacy, cost-effectiveness, etc.), while other submissions such as clinical guidelines and public health reviews were excluded. As the earliest reports dated back to the year 2000, we extracted HTA reports published from 2000 onwards.

To better demonstrate the current research progress on the HTA status of medical devices, we examined four case studies on medical devices, including (1) stents (2) hip and knee arthroplasty, (3) the da Vinci Surgical System, and (4) transcatheter aortic valve implantation (TAVI) and mitral valve repair (TMVR). We did not intend for the case studies to be representative of all medical devices as there is a great deal of diversity in medical devices beyond those four cases, such as diagnostic or instrumental devices. Instead, through our choice of target devices, we aimed to cover a range of heterogeneous cases in terms of disease epidemiology, procedure characteristics, technology maturity, and demographics. We used the number of HTA-related publications to measure the activity level of the current HTA research. We conducted a literature search and tracked the growth trend of relevant HTA publications on PubMed, Embase, and Web of Science. We included HTA studies and economic evaluations and excluded relevant systematic reviews or meta-analyses. The detailed search strategy in each database is listed in Additional file 1: Table S1.

In addition, a narrative literature review was conducted for a synthesis of the characteristic differences and challenges of HTA in Medical Devices. The literature search was performed using PubMed, Embase, and Web of Science. We included relevant empirical studies or reviews discussing the use of HTA for medical devices. The detailed search strategy in each database is listed in Additional file 1: Table S2.

Two reviewers (J.M. and Y.H.) independently assessed the titles and abstracts of all identified study and then reviewed full text to determine the potential eligibility for the above narrative literature review. Disagreements on whether a specific study should be considered were resolved by a third investigator (X.Z.).

To guide the efficient application of HTAs, we performed a gray literature search of official websites of major HTA agencies to identify HTA guidelines with respect to medical devices. As guidelines represent a consensus in the academic community, we believed that international HTA guidelines have reflected, to some extent, the current best possible practice. We complemented the search by reviewing the bibliographies of relevant literature identified through a target literature review of methodological publications on the HTA of medical devices. Only those (either guidelines or articles) that were specific to medical devices and elaborate economic evaluation, decision-analytic modeling, and/or HTA were included.

## Current status of the HTA of medical devices

### Published reports from HTA bodies

**Fig. 1:**
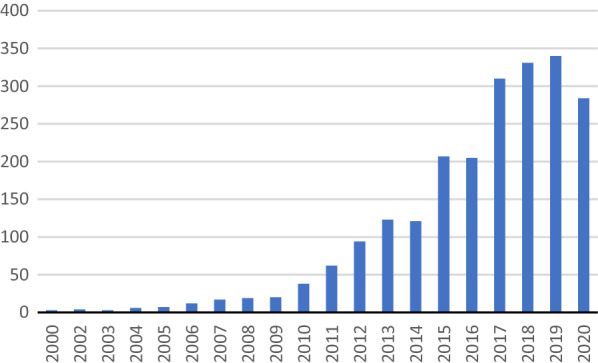
Number of Health Technology Assessment (HTA) reports for medical devices by country-year: 2000–2020

In total, around 2300 HTA reports from agencies across 30 countries or regions were identified. We presents the overall trend of HTA report submissions in Fig. 1. Overall, the body of HTA reports for medical devices increased across the world. Before 2010, the number of HTA reports published for medical devices was limited, ranging from three in 2000 to 20 in 2009. Since 2011, the number of published HTA reports has increased rapidly to reach 340 reports in 2019. Within the last 20 years, there has been a 100-fold increase in the number of HTA reports for medical devices.

### Journal publications on HTA of medical devices

**Fig. 2:**
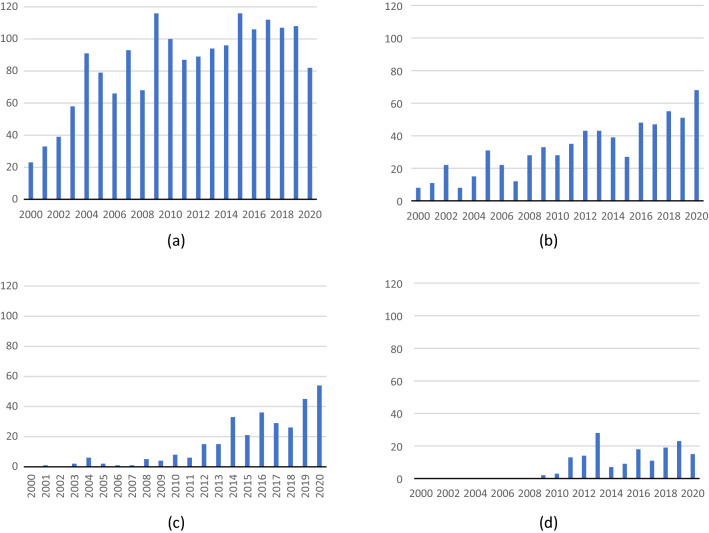
Summary of HTA-related literature. **a** Annual number of HTA-related publications on stents; **b** annual number of HTA-related publications on hip and knee arthroplasty; **c** annual number of HTA-related publications on Da Vinci; **d** annual number of HTA-related publications on TAVI and TMVR

Figure 2 shows the growth trend of HTA-related publications on the four selected devices, respectively. Overall, we observed a general upward trend in the four products, despite annual fluctuations, indicating that the HTA of medical devices has been growing rapidly. In addition, the level of development of HTA was also related to the characteristics of the medical device, such as technology maturity and disease epidemiology. We observed from Fig. 2a that there was a larger body of publications on stents compared to other devices since the stent was a mature device with broader applicable patient populations, indications, and long years of availability. In a comparison, TAVI and TMVR, as a relatively new product, had fewer relevant HTA publications. Additionally, there was significant growth in HTA publications for all four devices since their first market launch. The overall trend in relevant publications suggested a progressive increase in the HTA publications and academic interest in medical devices.

## Narrative synthesis of characteristic differences and challenges of HTA of medical devices

After a literature search on journal publications discussing HTA of medical devices, a total of 1646 records were identified, and 26 publications were included in our review after title and abstract screening or full text review. The PRISMA flowchart of literature review is provided in Additional file 1: Fig. S1.

The characteristic differences and challenges of HTA in medical devices are summarized in Table 1. Overall, there were several key characteristic differences between drugs and medical devices, including the availability of treatment outcomes and other factors that may impact efficacy. First, the treatment outcome for medical devices was not as clear and straightforward as it would be with drugs, because an intervention with device involves the medical devices themselves as well as other subsequent treatments. Furthermore, devices usually had multiple applications, making it hard to assess each application in the same way that traditional drugs were assessed for an individual indication. Second, Randomized Controlled Trials (RCTs) for medical devices are rare compared to drugs, resulting in a lack of sufficient efficacy/effectiveness data and making it difficult for economic evaluation. Third, the product life cycle of medical devices was generally much shorter than that of drugs, which may result in multiple specifications within a single product class and unclear definition of standard of care. Additionally, the efficacy of medical device treatments depends on the medical devices themselves and their use.

**Table 1:** Summary of characteristic differences and challenges of HTA of medical devices

Characteristic differences and challenges	Description	References
Available clinical evidence	The characteristic differences between pharmaceuticals and medical devices may lead to a large gap in terms of the availability of evidence, especially clinical evidence • Considerable challenges exist in performing an RCT for medical devices. For example, a double-blind procedure was usually hard to implement due to differences in the appearance of medical devices • Another reason was that implantable medical devices require informed consent from a patient before implantation as they involve an invasive procedure • There was a lack of infrastructure, e.g., qualified clinical centers and trained professionals to conduct RCTs for medical devices	[3–23]
Device-user interaction	Unlike drugs, the performance of medical devices sometimes depends on their users’ experience as well • The so-called “learning curve”: the launch of a medical device is followed by a training or initiation period during which healthcare professionals learn how to handle the technology. As healthcare professionals gain more experience over time, they were able to grasp the subtle differences that affected the overall clinical benefits, thus making the best use of the technology • The learning curve had inevitably interfered with the HTA of medical devices because the comparative effectiveness between newly-launched and traditional products was a function of the product itself and operators’ proficiency, which was hard to quantify • The clinical adoption of medical devices may also associate with wider impact of organizational change, for instance, there may be a need for additional training of physicians or other health professionals, or the introduction of a given device may require a hospital to reorganize services to accommodate the new technology or procedure	[3–9, 11–27]
Short product life cycle and quick upgrade	Unlike drugs, the product life cycle of medical devices is usually as short as one to 3 years • The key contributing factor was that medical devices undergo continuous improvement and incremental innovation, which might result in the existence of various models and specifications within a single product class • Recognizing the iterative nature of medical devices, regulatory agencies exempted such variants from rigorous clinical trials as long as the safety of the new variant was the same as the original. As a result, manufacturers had neither enough time to collect data for economic evaluations nor an incentive to invest in clinical research and HTA • From researchers’ perspective, the short life cycle required the HTA of medical devices to be done in a timely manner, otherwise the results could become outdated • Pricing was typically more dynamic than that of pharmaceuticals, which increased the complexity of calculating costs • Rapid product iteration also made it difficult to conduct HTA since the definition of standard of care was unclear or constantly changing among medical devices with multiple specifications and models	[4–9, 11–20, 22, 25–28]
Inexplicit target population and lack of direct clinical outcomes	The economic evaluation of medical devices used in screening or diagnostics was ever more challenging as most did not have an explicit target population, nor do they produce clinical outcomes directly • These devices were used in multiple disease areas or as part of the care pathway with a group of other devices. For example, the positron emission tomography-computed tomography (PET/CT) was used in the diagnosis and follow-up of a number of malignant tumors (e.g., cervical cancer, colorectal cancer, non-small cell lung cancer, melanoma, and ovarian cancer) • Since these devices or diagnostics are integrated as a specific part of the clinical pathway. As a result, it is hard to quantitatively observe the direct impact of these medical devices on patients’ ultimate medical outcomes	[3, 20, 23, 24, 27]

## Discussions on practical solutions for the challenges of HTA of medical devices

We obtained a total of eight HTA guidelines specific to medical devices issued by HTA agencies or research initiatives across six regions. The National Institute for Health and Care Excellence (NICE) in the United Kingdom issued an HTA methods guide for their Medical Technologies Evaluation Programme in 2011 [29]. Following the methods guide, NICE also issued the Diagnostics Assessment Programme manual specifically for diagnostic technologies demonstrating higher test accuracy, but at a greater cost compared to those in current use [30]. In Canada, Health Quality Ontario (HQO) released a method and process guide for HTA in 2018, with a scope spanning from medical devices, diagnostics, and surgical procedures to complex health system interventions [31]. In Australia, two HTA guidelines have been developed separately for therapeutic and diagnostic devices by the Medical Services Advisory Committee (MSAC) [32, 33]. In the Asia–Pacific region, the Singapore Agency for Care Effectiveness (ACE) was the only national HTA organization that has released HTA guidelines on medical devices [34]. Apart from these official HTA agencies, an international collaborative network also contributed to the methodological advancement of HTA for medical devices. For example, the European Network for Health Technology Assessment (EUnetHTA), has launched a series of research initiatives to develop a methodological framework for HTA of therapeutic medical devices [35].

### Available clinical evidence

Given that RCT evidence for medical devices was generally limited, an open-minded and flexible attitude to other forms of evidence e.g., case reports (series), cohort studies, case control studies, and real-world studies was highly recommended [29, 34, 35]. Both the UK and EUnetHTA guidelines have pointed out the high risk of bias in non-randomized controlled trials [30, 35]. At the same time, several tools have been developed, although they may not be specific for medical devices. The Cochrane Risk of Bias Assessment Tool for Non-Randomized Studies of Interventions (ACROBAT-NRSI) could be used to assess the risk of bias in non-randomized controlled studies [36]. In addition, the quality assessment for case reports (series) could refer to the checklist developed by the Canadian Institute of Health Economics [37].

The draft guidance released by the United States Food and Drug Administration (FDA) in 2016 has spurred a surge in the literature describing how real-world evidence (RWE) can be used to support regulatory approval for medical devices [38]. RWE refers to any evidence on healthcare generated from multiple sources outside clinical trial settings, which is usually in the form of electronic medical records (EMR), electronic health records (EHR), hospital databases, patient registries, claims data, etc. [39]. In addition to market authorization, RWE was also relevant in post-marketing surveillance, coverage decisions, outcome-based contracting, resource use, and treatment compliance [40, 41]. Especially for medical device products for which the regulatory environment does not require RCTs, or in situations where RCTs traditionally have been lacking such as measuring disease burden and detecting new safety signals, RWE could offer unique perspectives.

Unlike randomized clinical trials, most RWE comes from observational studies and might have many drawbacks. While current medical device-specific HTA guidelines have underscored the potential bias associated with RWE and several tools may be available for assessment of bias for non-randomized studies, few guidelines have addressed other common issues including data quality, availability, standards, and privacy [29, 30, 32, 33, 35, 42]. For example, a European study that mapped RWE studies of three medical device products has revealed that the accessibility of data sources for RWE varied greatly across European countries. The study also suggested the types and definitions of variables included in each data source were not consistent, making a comparison across databases impossible [43]. Therefore, there is a need for RWE guidance on medical devices which would not only provide overarching frameworks but also standardize methods and processes ranging from data storage, collection, and sharing to analytic approaches.

### Device–user interaction

International medical device-specific HTA guidelines have emphasized the need to account for the learning curve effect in HTA. The EUnetHTA has suggested that it is necessary to establish a break-in period before the formal evaluation to ensure that users have sufficient time to adapt to the new technology. Also, various degrees of operator proficiency across different types of medical research centers (e.g., teaching hospitals and non-teaching hospitals) would lead to heterogeneity in HTA. Therefore, the EUnetHTA proposed a three-tiered approach to accounting for the learning curve in its HTA guidelines for therapeutic devices. Firstly, assessors should screen for studies that estimate an association between user proficiency or healthcare settings (e.g., teaching or non-teaching hospitals) and clinical outcomes. Secondly, if the effect of the learning curve was not reported in the RCT and relevant information could not be obtained by contacting the investigators, then other types of evidence such as non-randomized controlled and non-comparative effectiveness studies could also be considered in order to explore the association between operator proficiency, types of study centers, and clinical outcomes. Lastly, subgroup analyses could be applied where existing studies were divided into different subgroups based on the level of operator proficiency. Statistical methods such as meta-analysis could be used to estimate the difference in medical outcomes between these subgroups and hence quantify the effect of the learning curve [35]. The radiofrequency ablation (RFA) for liver tumors treatment serves as an example. In a systematic review, researchers divided 100 case reports into four subgroups according to the surgeons’ previous RFA experience (i.e., having done < 20, 21–50, 51–99, > 100 cases respectively). The results of the meta-analysis showed the tumor recurrence rate decreased (18%, 16%, 14%, and 10% respectively in the four subgroups) as surgeons accumulated experience [44].

### Short product life cycle and quick upgrade

In practice, a Bayesian approach was recommended to account for the iterative nature of medical devices in HTA [35]. The Bayesian approach is a statistical method that infers the posterior distribution of unknown parameters according to Bayes’ theorem based on prior knowledge and sample data. Considering that medical devices are incrementally upgraded with minor modifications, clinical trials and/or early research data of the former version of the medical device product, sometimes even data of comparator products could be a source for prior information used in the Bayesian approach.

### Inexplicit target population and lack of direct clinical outcomes

Given the lack of direct clinical outcomes for screening and diagnostic devices, the HQO allows the use of established surrogate endpoints or intermediate clinical indicators to predict patients’ final medical outcomes. For instance, the association between intermediate indicators (e.g., blood pressure) and cardiovascular-related deaths has already been established through statistical models [31]. In terms of evaluating screening or diagnostic technologies, NICE, MSAC, and EUnetHTA stress that product performance should be reflected in the entire care pathway. In this way, the HTA should not only evaluate the test accuracy, but also consider the impact of the diagnostic results (no matter how accurate they were) on subsequent treatment pathways and the final medical outcomes [30, 32, 35]. One particular technique described by international HTA guidelines is the linked analysis [30, 32]. In its first step, a linked analysis collects comprehensive data on the test accuracy of diagnostic technologies and the effectiveness of subsequent clinical interventions following the diagnostic results. Then, these data are modeled to simulate the whole care pathway and to estimate the impact of the diagnostic device on the final medical outcome [30]. However, it is worth mentioning that there were two premises for conducting linked analysis: (1) the effectiveness of clinical interventions subsequent to the diagnostic results must be established by confirmatory trials and should be available; (2) Patients’ baseline characteristics in these confirmatory trials of the subsequent clinical interventions should resemble the population to which the diagnostic devices were applied.

## Prospects

### Considering multi-source evidence such as real-world evidence

Most HTAs of pharmaceuticals have been performed using economic evaluations with parameters derived from RCTs. However, the market authorization for most medical devices does not require rigorous RCTs, leading to limited clinical evidence. The scarcity of clinical research has made RWE particularly important in generating clinical effectiveness and safety data for the HTA of medical devices. Unlike the ideal experimental environment of RCTs, the “real world” refers to actual clinical settings where patients have not been selected based on pre-specified criteria. Patients enrolled in RWE studies tend to cover different subgroups so that they are representative of the whole population. For this reason, RWE reflects the true effects of clinical interventions. Correspondingly, HTA based on RWE could provide healthcare decision-makers with insights that came from real-world settings. As the uptake of newly introduced medical devices often requires a break-in period, this creates the perfect timing to collect real-world data on products’ safety and effectiveness. In addition to RWE, HTA could also collect public opinions from multiple third parties (patients, manufacturers, health care providers) regarding current evidence, treatment pattern, and patient categories.

### Standardization of tools and evaluation criteria for HTA of medical devices

Existing HTA guidelines mainly focus on drugs and cannot be applied directly to the HTA of medical devices even with adaptation. Therefore, we suggest that separate HTA guidelines for medical devices are needed to standardize the topic identification, selection of comparator, evaluation methods, cost measurement, effect/utility measurement, evidence synthesis, systemic review, and ethnic requirements. Moreover, the HTA report should follow a consistent reporting paradigm. We also recommend that decision-makers follow the same HTA guidelines to conduct HTA appraisals. The formulation of HTA guidelines should be transparent and publicly available. At the same time, regular updates are necessary to reflect the evolution of HTA methods, and international collaboration is needed in overcoming the inherent challenges in medical device HTA.

### Intergration of HTA of medical devices into decision-making

As a bridge connecting scientific research and health decision-making, the development of HTA is closely interwoven with established mechanisms such that the results of HTA could be translated into real practice. HTA as well as value assessment methods have been adopted around the world in national coverage decisions for pharmaceuticals. Nevertheless, the application of HTA in medical devices decision-making is in an earlier stage with higher uncertainty. Therefore, it is essential to explore an effective mechanism that would enable the translation of the results of HTA of medical devices into decision-making. Specifically, the decision translation mechanism could take the form of regulatory authorization, market access and reimbursement, and price negotiations where HTA could be introduced. We believe that better integration of HTA into decision-making would further encourage evidence generation and the adoption of HTA standards and ultimately promote an evidence-based, decision-making culture.

## Conclusion

The body of HTA reports and journal publications on medical devices around the world has been growing. Our analysis revealed that medical devices differ considerably from pharmaceuticals in many respects, which has made the HTA of medical devices quite challenging. These challenges include scarcity of well-designed RCTs, inconsistent RWE data sources and methods, device-user interaction, short product lifecycle, inexplicit target population, and lack of direct medical outcomes.

Practical solutions found in the HTA guidelines to account for these challenges include (1) adopting an open mind toward evidence other than that generated through an RCT, such as RWE, especially as newly introduced medical devices often require a break-in period; (2) accounting for the learning curve that impacts the device-user interaction through several means including subgroup analyses; (3) applying a Bayesian approach to account for the iterative nature of medical devices; and (4) ensuring that product performance is measured across the entire care pathway through techniques such as linked analyses.

Based on the results of the above analysis, we call on both academic communities and relevant agencies to standardize the process, methodologies, and criteria of HTAs on medical devices, particularly when an HTA has involved RWE studies. We also recommend that national authorities better integrate the HTA of medical devices into decision-making and promote a more evidence-based culture.

## Supplementary Information

**Additional file 1: Table S1.** Search strategy of journal publications on HTA of selected medical devices. **Table S2.** Search strategy of journal publications on discussing HTA of medical devices. **Figure S1.** PRISMA flowchart of literature review of journal publications on discussing HTA of medical devices.

## Data Availability

The data used and/or analyzed during the study are available from the corresponding author on reasonable request.
