# Supplementary material for: Health technology assessment of medical devices: current landscape, challenges, and a way forward
Source: Cost Eff Resour Alloc. 2022 Oct 5;20:54. doi: 10.1186/s12962-022-00389-6 (PMC9533595; doi:10.1186/s12962-022-00389-6)
Supplement: Supplementary file 1 — Additional file 1: Table S1. Search strategy of journal publications on HTA of selected medical devices. Table S2. Search strategy of journal publications on discussing HTA of medical devices. Figure S1. PRISMA flowchart of literature review of journal publications on discussing HTA of medical devices. [file 12962_2022_389_MOESM1_ESM.docx]

Health Technology Assessment of Medical Devices: Current Landscape, Challenges, and a Way Forward

**Additional Materials**

**Table S1 Search strategy of journal publications on HTA of selected medical devices**

| **Medical devices** | **Database** | **Search strategy** |
| --- | --- | --- |
| **Hip and knee arthroplasty** | **PubMed** | (("cost-effectiveness analysis"[Title/Abstract] OR "cost effectiveness analysis"[Title/Abstract] OR "cost-benefit analysis"[Title/Abstract] OR "cost benefit analysis"[Title/Abstract] OR "cost-minimization analysis"[Title/Abstract] OR "cost minimization analysis"[Title/Abstract] OR "cost-utility analysis"[Title/Abstract] OR "cost utility analysis"[Title/Abstract] OR "incremental cost-effectiveness ratio"[Title/Abstract] OR "incremental cost effectiveness ratio"[Title/Abstract] OR "ICER"[Title/Abstract] OR "CEA"[Title/Abstract] OR "CBA"[Title/Abstract] OR "CMA"[Title/Abstract] OR "CUA"[Title/Abstract] OR "health technology assessment"[Title/Abstract] OR "economic evaluation"[Title/Abstract]) AND ("Arthrosis"[Title/Abstract] OR replacement[Title/Abstract] OR revision[Title/Abstract] OR endoprosthesis[Title/Abstract]) AND (knee*[Title/Abstract] OR hip[Title/Abstract])) NOT (meta-analysis[Filter] OR review[Filter] OR systematicreview[Filter]) |
|  | **Embase** | 1 cost-effectiveness analysis.ab,ti.  2 cost-benefit analysis.ab,ti.  3 cost-minimization analysis.ab,ti.  4 cost-utility analysis.ab,ti.  5 incremental cost-effectiveness ratio.ab,ti.  6 cost effectiveness analysis.ab,ti.  7 cost benefit analysis.ab,ti.  8 cost minimization analysis.ab,ti.  9 cost utility analysis.ab,ti.  10 incremental cost effectiveness ratio.ab,ti.  11 CEA.ab,ti.  12 CBA.ab,ti.  13 CMA.ab,ti.  14 CUA.ab,ti.  15 ICER.ab,ti.  16 Health technology assessment.ab,ti.  17 Economic evaluation.ab,ti.  18 1 or 2 or 3 or 4 or 5 or 6 or 7 or 8 or 9 or 10 or 11 or 12 or 13 or 14 or 15 or 16 or 17  19 ((arthrosis or replacement or revision or endoprosthesis) adj2 (knee* or hip)).ab,ti.  20 18 and 19  21 limit 20 to (meta analysis or “systematic review”)  22 20 not 21 |
|  | **Web of science** | 1 TI=(cost-effectiveness analysis OR cost effectiveness analysis OR cost-benefit analysis OR cost benefit analysis OR cost-minimization analysis OR cost minimization analysis OR cost-utility analysis OR cost utility analysis OR incremental cost-effectiveness ratio OR incremental cost effectiveness ratio OR CEA OR CBA OR CUA OR ICER OR economic evaluation OR health technology assessment) AND TI=(Arthrosis OR replacement OR revision OR endoprosthesis) AND TI=(knee* OR hip)  2 AB=(cost-effectiveness analysis OR cost effectiveness analysis OR cost-benefit analysis OR cost benefit analysis OR cost-minimization analysis OR cost minimization analysis OR cost-utility analysis OR cost utility analysis OR incremental cost-effectiveness ratio OR incremental cost effectiveness ratio OR CEA OR CBA OR CUA OR ICER OR economic evaluation OR health technology assessment) AND AB=(Arthrosis OR replacement OR revision OR endoprosthesis) AND AB=(knee* OR hip)  3 #1 or #2  4 Refined by: [excluding] DOCUMENT TYPES: (REVIEW) |
| **TAVI and TMVR** | **PubMed** | (("cost-effectiveness analysis"[Title/Abstract] OR "cost effectiveness analysis"[Title/Abstract] OR "cost-benefit analysis"[Title/Abstract] OR "cost benefit analysis"[Title/Abstract] OR "cost-minimization analysis"[Title/Abstract] OR "cost minimization analysis"[Title/Abstract] OR "cost-utility analysis"[Title/Abstract] OR "cost utility analysis"[Title/Abstract] OR "incremental cost-effectiveness ratio"[Title/Abstract] OR "incremental cost effectiveness ratio"[Title/Abstract] OR "ICER"[Title/Abstract] OR "CEA"[Title/Abstract] OR "CBA"[Title/Abstract] OR "CMA"[Title/Abstract] OR "CUA"[Title/Abstract] OR "health technology assessment"[Title/Abstract] OR "economic evaluation"[Title/Abstract]) AND ("TransCatheter Valve Treatment"[Title/Abstract] OR "Transcatheter Aortic Valve Implantation"[Title/Abstract] OR "TAVI"[Title/Abstract] OR "Transcatheter Mitral Valve Repair"[Title/Abstract] OR "TMVR"[Title/Abstract])) NOT (meta-analysis[Filter] OR review[Filter] OR systematicreview[Filter]) |
|  | **Embase** | 1 cost-effectiveness analysis.ab,ti.  2 cost-benefit analysis.ab,ti.  3 cost-minimization analysis.ab,ti.  4 cost-utility analysis.ab,ti.  5 incremental cost-effectiveness ratio.ab,ti.  6 cost effectiveness analysis.ab,ti.  7 cost benefit analysis.ab,ti.  8 cost minimization analysis.ab,ti.  9 cost utility analysis.ab,ti.  10 incremental cost effectiveness ratio.ab,ti.  11 CEA.ab,ti.  12 CBA.ab,ti.  13 CMA.ab,ti.  14 CUA.ab,ti.  15 ICER.ab,ti.  16 Health technology assessment.ab,ti.  17 Economic evaluation.ab,ti.  18 1 or 2 or 3 or 4 or 5 or 6 or 7 or 8 or 9 or 10 or 11 or 12 or 13 or 14 or 15 or 16 or 17  19 TransCatheter Valve Treatment.ab,ti.  20 Transcatheter Aortic Valve Implantation.ab,ti.  21 TAVI.ab,ti.  22 Transcatheter Mitral Valve Repair.ab,ti.  23 TMVR.ab,ti  24 19 or 20 or 21 or 22 or 23  25 18 and 24  26 limit 25 to (meta analysis or “systematic review”)  27 25 not 26 |
|  | **Web of science** | 1 TI=(cost-effectiveness analysis OR cost effectiveness analysis OR cost-benefit analysis OR cost benefit analysis OR cost-minimization analysis OR cost minimization analysis OR cost-utility analysis OR cost utility analysis OR incremental cost-effectiveness ratio OR incremental cost effectiveness ratio OR CEA OR CBA OR CUA OR ICER OR economic evaluation OR health technology assessment) AND TI=( TransCatheter Valve Treatment OR Transcatheter Aortic Valve Implantation OR TAVI OR Transcatheter Mitral Valve Repair OR TMVR)  2 AB=(cost-effectiveness analysis OR cost effectiveness analysis OR cost-benefit analysis OR cost benefit analysis OR cost-minimization analysis OR cost minimization analysis OR cost-utility analysis OR cost utility analysis OR incremental cost-effectiveness ratio OR incremental cost effectiveness ratio OR CEA OR CBA OR CUA OR ICER OR economic evaluation OR health technology assessment) AND AB=( TransCatheter Valve Treatment OR Transcatheter Aortic Valve Implantation OR TAVI OR Transcatheter Mitral Valve Repair OR TMVR)  3 #1 or #2  4 Refined by: [excluding] DOCUMENT TYPES: (REVIEW) |
| **The da Vinci Surgical System** | **PubMed** | (("cost-effectiveness analysis"[Title/Abstract] OR "cost effectiveness analysis"[Title/Abstract] OR "cost-benefit analysis"[Title/Abstract] OR "cost benefit analysis"[Title/Abstract] OR "cost-minimization analysis"[Title/Abstract] OR "cost minimization analysis"[Title/Abstract] OR "cost-utility analysis"[Title/Abstract] OR "cost utility analysis"[Title/Abstract] OR "incremental cost-effectiveness ratio"[Title/Abstract] OR "incremental cost effectiveness ratio"[Title/Abstract] OR "ICER"[Title/Abstract] OR "CEA"[Title/Abstract] OR "CBA"[Title/Abstract] OR "CMA"[Title/Abstract] OR "CUA"[Title/Abstract] OR "health technology assessment"[Title/Abstract] OR "economic evaluation"[Title/Abstract]) AND ("Da Vinci" OR "Davinci" OR "robotic surgery" OR "robot surgery" OR "robotic surgeries")) NOT (meta-analysis[Filter] OR review[Filter] OR systematicreview[Filter]) |
|  | **Embase** | 1 cost-effectiveness analysis.ab,ti.  2 cost-benefit analysis.ab,ti.  3 cost-minimization analysis.ab,ti.  4 cost-utility analysis.ab,ti.  5 incremental cost-effectiveness ratio.ab,ti.  6 cost effectiveness analysis.ab,ti.  7 cost benefit analysis.ab,ti.  8 cost minimization analysis.ab,ti.  9 cost utility analysis.ab,ti.  10 incremental cost effectiveness ratio.ab,ti.  11 CEA.ab,ti.  12 CBA.ab,ti.  13 CMA.ab,ti.  14 CUA.ab,ti.  15 ICER.ab,ti.  16 Health technology assessment.ab,ti.  17 Economic evaluation.ab,ti.  18 1 or 2 or 3 or 4 or 5 or 6 or 7 or 8 or 9 or 10 or 11 or 12 or 13 or 14 or 15 or 16 or 17  19 robotic surgery.ab,ti.  20 robot surgery.ab,ti.  21 robotic surgeries.ab,ti.  22 Da Vinci.ab,ti.  23 Davinci.ab,ti.  24 19 or 20 or 21 or 22 or 23  25 18 and 24  26 limit 25 to (meta analysis or “systematic review”)  27 25 not 26 |
|  | **Web of science** | 1 TI=(cost-effectiveness analysis OR cost effectiveness analysis OR cost-benefit analysis OR cost benefit analysis OR cost-minimization analysis OR cost minimization analysis OR cost-utility analysis OR cost utility analysis OR incremental cost-effectiveness ratio OR incremental cost effectiveness ratio OR CEA OR CBA OR CUA OR ICER OR economic evaluation OR health technology assessment) AND TI=(robotic surgery OR robot surgery OR robotic surgeries OR Da Vinci OR Davinci)  2 AB=(cost-effectiveness analysis OR cost effectiveness analysis OR cost-benefit analysis OR cost benefit analysis OR cost-minimization analysis OR cost minimization analysis OR cost-utility analysis OR cost utility analysis OR incremental cost-effectiveness ratio OR incremental cost effectiveness ratio OR CEA OR CBA OR CUA OR ICER OR economic evaluation OR health technology assessment) AND AB=(robotic surgery OR robot surgery OR robotic surgeries OR Da Vinci OR Davinci)  3 #1 or #2  4 Refined by: [excluding] DOCUMENT TYPES: (REVIEW) |
| **stent** | **PubMed** | (("cost-effectiveness analysis"[Title/Abstract] OR "cost effectiveness analysis"[Title/Abstract] OR "cost-benefit analysis"[Title/Abstract] OR "cost benefit analysis"[Title/Abstract] OR "cost-minimization analysis"[Title/Abstract] OR "cost minimization analysis"[Title/Abstract] OR "cost-utility analysis"[Title/Abstract] OR "cost utility analysis"[Title/Abstract] OR "incremental cost-effectiveness ratio"[Title/Abstract] OR "incremental cost effectiveness ratio"[Title/Abstract] OR "ICER"[Title/Abstract] OR "CEA"[Title/Abstract] OR "CBA"[Title/Abstract] OR "CMA"[Title/Abstract] OR "CUA"[Title/Abstract] OR "health technology assessment"[Title/Abstract] OR "economic evaluation"[Title/Abstract]) AND (“stent”[Title/Abstract])) NOT (meta-analysis[Filter] OR review[Filter] OR systematicreview[Filter]) |
|  | **Embase** | 1 cost-effectiveness analysis.ab,ti.  2 cost-benefit analysis.ab,ti.  3 cost-minimization analysis.ab,ti.  4 cost-utility analysis.ab,ti.  5 incremental cost-effectiveness ratio.ab,ti.  6 cost effectiveness analysis.ab,ti.  7 cost benefit analysis.ab,ti.  8 cost minimization analysis.ab,ti.  9 cost utility analysis.ab,ti.  10 incremental cost effectiveness ratio.ab,ti.  11 CEA.ab,ti.  12 CBA.ab,ti.  13 CMA.ab,ti.  14 CUA.ab,ti.  15 ICER.ab,ti.  16 Health technology assessment.ab,ti.  17 Economic evaluation.ab,ti.  18 1 or 2 or 3 or 4 or 5 or 6 or 7 or 8 or 9 or 10 or 11 or 12 or 13 or 14 or 15 or 16 or 17  19 stent.ab,ti.  20 18 and 19  21 limit 20 to (meta analysis or “systematic review”)  22 20 not 21 |
|  | **Web of science** | 1 TI=(cost-effectiveness analysis OR cost effectiveness analysis OR cost-benefit analysis OR cost benefit analysis OR cost-minimization analysis OR cost minimization analysis OR cost-utility analysis OR cost utility analysis OR incremental cost-effectiveness ratio OR incremental cost effectiveness ratio OR CEA OR CBA OR CUA OR ICER OR economic evaluation OR health technology assessment) AND TI=(stent)  2 AB=(cost-effectiveness analysis OR cost effectiveness analysis OR cost-benefit analysis OR cost benefit analysis OR cost-minimization analysis OR cost minimization analysis OR cost-utility analysis OR cost utility analysis OR incremental cost-effectiveness ratio OR incremental cost effectiveness ratio OR CEA OR CBA OR CUA OR ICER OR economic evaluation OR health technology assessment) AND AB=(stent)  3 #1 or #2  4 Refined by: [excluding] DOCUMENT TYPES: (REVIEW) |

**Table S2 Search strategy of journal publications on discussing HTA of medical devices**

| **Medical devices** | **Database** | **Search strategy** | **Results number** |
| --- | --- | --- | --- |
| **HTA of medical devices** | **Pubmed** | ("health technology assessment"[Title/Abstract] OR "economic evaluation"[Title/Abstract] OR "Pharmacoeconomics"[Title/Abstract] OR "HTA"[Title/Abstract]) AND ("medical device"[Title/Abstract] OR "medical devices"[Title/Abstract]) | 242 |
|  | **Embase** | 1 HTA.ab. or HTA.ti. or economic evaluation.ab. or economic evaluation.ti. or pharmacoeconomic.ab. or pharmacoeconomic.ti. or health technology assessment.ab. or health technology assessment.ti.  2 medical device.ab. or medical device.ti. or medical devices.ab. or medical devices.ti.  3 1 and 2 | 456 |
|  | **Web of Science** | 1 TI=(HTA OR economic evaluation OR pharmacoeconomic OR health technology assessment) AND TI=(medical device OR medical devices)  2 AB=(HTA OR economic evaluation OR Pharmacoeconomics OR health technology assessment) AND AB=(medical device OR medical devices)  3 #1 or #2 | 948 |


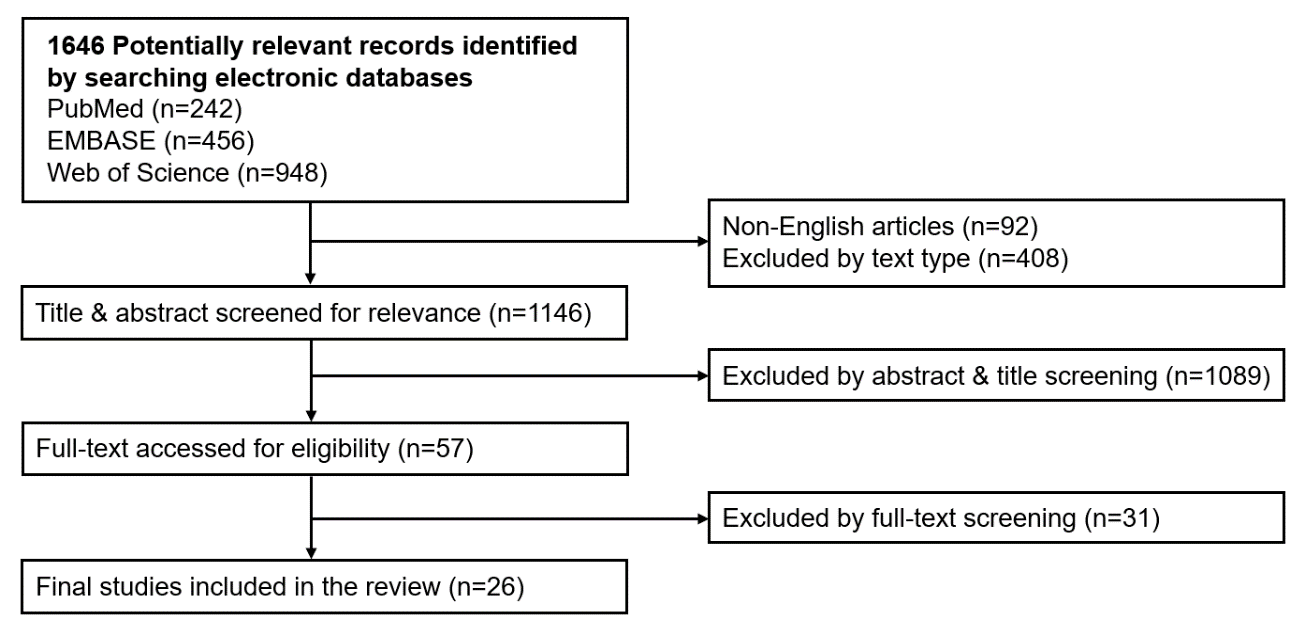


**Figure S1: PRISMA flowchart of literature review of journal publications on discussing HTA of medical devices**
